# Acupressure for smoking cessation – a pilot study

**DOI:** 10.1186/1472-6882-7-8

**Published:** 2007-03-14

**Authors:** Adrian R White, Russell C Moody, John L Campbell

**Affiliations:** 1 Primary Care Research Group, Peninsula Medical School, Universities of Exeter and Plymouth, UK; 2 Training and Development Co-ordinator, The Smoking Advice Service, Nuffield Clinic Lipson Rd, Plymouth, UK

## Abstract

**Background:**

Tobacco smoking is a serious risk to health: several therapies are available to assist those who wish to stop. Smokers who approach publicly funded stop-smoking clinics in the UK are currently offered nicotine replacement therapy (NRT) or bupropion, and group behaviour therapy, for which there is evidence of effectiveness. Acupuncture and acupressure are also used to help smokers, though a systematic review of the evidence of their effectiveness was inconclusive. The aim of this pilot project was to determine the feasibility of a study to test acupressure as an adjunct to one anti-smoking treatment currently offered, and to inform the design of the study.

**Methods:**

An open randomised controlled pilot study was conducted within the six week group programme offered by the Smoking Advice Service in Plymouth, UK. All participants received the usual treatment with NRT and group behavioural therapy, and were randomised into three groups: group A with two auricular acupressure beads, group B with one bead, and group C with no additional therapy. Participants were taught to press the beads when they experienced cravings. Beads were worn in one ear for four weeks, being replaced as necessary. The main outcome measures assessed in the pilot were success at quitting (expired CO ≤ 9 ppm), the dose of NRT used, and the rating of withdrawal symptoms using the Mood and Symptoms Scale.

**Results:**

From 49 smokers attending four clinics, 24 volunteered to participate, 19 attended at least once after quitting, and seven remained to the final week. Participants who dropped out reported significantly fewer previous quit attempts, but no other significant differences. Participants reported stimulating the beads as expected during the initial days after quitting, but most soon reduced the frequency of stimulation. The discomfort caused by the beads was minor, and there were no significant side effects. There were technical problems with adhesiveness of the dressing. Reporting of NRT consumption was poor, with much missing data, but reporting of ratings of withdrawal symptom scores was nearly complete. However, these showed no significant changes or differences between groups for any week.

**Conclusion:**

Any effects of acupressure on smoking withdrawal, as an adjunct to the use of NRT and behavioural intervention, are unlikely to be detectable by the methods used here and further preliminary studies are required before the hypothesis can be tested.

## Background

Smoking is recognised as a major cause of ill health, and in developed countries cigarettes are the single biggest avoidable cause of death and disability [1], so that quitting smoking is generally the single most important action that any individual can take to improve health [2]. But stopping smoking is known to be difficult, mainly because of nicotine withdrawal symptoms. Most smokers wish they were non-smokers, but their attempts to stop frequently fail [3]. The unassisted quit rate at one year is about 3% [4], and a wide range of interventions have been proposed to help those who want to quit.

One such intervention is acupuncture, which has been used for centuries in China for treating opium withdrawal symptoms [5]. Acupuncture using only points in the ear (auricular acupuncture) was found by chance to help withdrawal in opium smokers in Hong Kong in the 1970s [6]. One common technique of auricular acupuncture involves inserting an small indwelling needle in the ear, which is pressed by the wearer whenever cravings occur. The needle should be removed and replaced frequently to avoid infection.

There is some evidence that acupuncture may act on the nucleus accumbens to inhibit the rise in dopamine which seems to be the common pathway for withdrawal symptoms [7,8]. However, a systematic review of the acupuncture literature found that, overall, the evidence was inconclusive on whether acupuncture is clinically effective for nicotine withdrawal [9]. A subsequent review which explored the data for auricular acupuncture only suggested that this result might be a false negative [10]. Most controlled trials had compared the 'correct' sites with 'incorrect' sites on the ear, but there is some doubt whether any sites in the ear can be regarded as 'incorrect'. Auricular acupuncture was more effective than other interventions it was compared with, but not more effective than treatment at 'incorrect' sites.

Other interventions for smoking cessation have developed an evidence base, and there is now clear evidence to support pharmacological therapy with nicotine replacement therapy (NRT) or bupropion, to reduce withdrawal symptoms [4]; and psychological therapies including education, support, and behavioural therapy on either a group [11] or individual basis [12] to deal with the behavioural aspects of quitting. Some of these therapies may double the abstinence rate at one year [13]. They are now widely available in England as part of the NHS Stop Smoking Service set up in response to the Government's White Paper *Smoking Kills *[14]. Data available for the clinic in Plymouth show that over 300 patients attended courses of these interventions in 2003 (Smoking Advice Service report April 2004).

In the context of the success of NRT and counselling, it is reasonable to ask whether acupuncture has a role as a possible adjunct. There is some evidence that combining different methods for quitting increases success rates more than by simple addition [15]. It would be of interest if acupuncture could either further reduce the withdrawal symptoms and increase the success rate, or reduce the use of NRT and possibly minimise total costs of the intervention.

However, auricular acupuncture with indwelling needles is not without problems, particularly the risk of lost needles causing spread of blood borne infection. The major professional body of medical acupuncturists in the UK no longer recommends the use of indwelling needles [16]. Therefore small beads (acupressure) are commonly used as a substitute and one study found them superior to advice alone in smoking cessation [17]. We decided to conduct a pilot study of acupressure within the setting of the Stop Smoking Service, to explore the feasibility of conducting a full scale randomised controlled trial. We wished to address the questions of a) the accrual and dropout rates, b) compliance with stimulation of beads, c) adverse events. We also wished to explore the feasibility of the planned outcome measures, involving d) compliance with completing symptom diaries and e) methods to monitor the use of NRT. In addition, we sought f) information on outcome data variance and possible effect size, for sample size calculation.

## Methods

An open pilot RCT was conducted with three arms: two treatment groups with one or two acupressure beads, and a control group who received no acupressure. All participants received usual therapy. Approval was given by the South West Devon Research Ethics Committee (04/Q2103/154).

The study was run within the group therapy service of the community based, NHS-funded Smoking Advice Service in Plymouth. Smokers join a Quit Smoking Group which meets for six consecutive weeks (Table 1). At the initial meeting, smokers are offered general information and motivational and behavioural counselling, and given a letter to the GP recommending the dose of NRT or, more rarely, bupropion tailored to the individual according to their smoking history. The quit date is set for the second session one week later, when members of the group smoke their last cigarette and take their medication for the first time. Four subsequent weekly meetings offer monitoring, advice and group support.

**Table 1:** Study flow chart indicating the acupressure study running alongside the usual smoking clinics

clinic attendance	1	2	3	4	5	6
Smoking Advice Service activity	advice; prescription for nicotine replacement	QUIT DATE 1^st ^nicotine replacement	behavioural support & compliance	behavioural support & compliance	behavioural support & compliance	behavioural support & compliance
acupressure study activity	information, consent	groups A & B: bead(s) placed	replaced if necessary	replaced if necessary	replaced if necessary	removed
study diary issued	run-in	quit 1	quit 2	quit 3	quit 4	

Smokers were invited to join the study by letter which included the study information sheet and questionnaire, sent before their first meeting. At the first group session, we explained the research. Smokers were eligible for inclusion if they smoked ≥ 10 cigarettes/day, were aged 18 years or over, intended to stop smoking on the quit date, chose NRT rather than bupropion, and gave informed consent. Exclusion criteria were: history of current otitis externa or other pathological condition of the ear, history of a poorly controlled relevant medical condition, currently taking anti-depressant or anti-psychotic medication, history of allergy to adhesive dressing, belief of pregnancy, or already participating in a research project. All smokers wishing to join the study were interviewed on first attendance by AW or RM; they then gave consent and provided socio-demographic information and smoking history including the Fagerstrom severity questionnaire.

All participants in the study received the usual NRT and group counselling and support; on the quit date, participants opened an opaque, numbered envelope containing a code generated by computerised blocked randomisation (blocks of four) prepared by a researcher unconnected with the study. The three codes indicated A) two acupressure beads B) one bead, both in addition to the usual interventions, and C) usual interventions only.

### Acupressure interventions

In the intervention groups, beads were applied on the second attendance (quit day) by AW or RM. Beads were of Pyonex^® ^type, manufactured without a needle for this study (Seirin, Japan).

In group A, two beads were placed, using the so-called 'Lung' and 'Shenmen' points of the ear; in group B, one bead was placed at Lung point. These points are the two most commonly used in the literature [18] and included in a manual [19]. They were identified by inspection of surface anatomy, looking for the most prominent depression in the centre of the concha (Lung point), and the anterior angle of the triangular fossa (Shenmen). The dominant side of the body was used in the first instance. Participants were given oral and written instructions on how to tap, press or squeeze the beads repeatedly for up to a minute each time a craving occurs. They were also advised on maintaining local hygiene, and on possible adverse events and how to deal with them including how to remove the bead if it became painful. They were also asked to bring their empty packets of NRT to subsequent clinics, to allow direct assessment of NRT consumption. All participants' GPs were informed by letter of their participation in the trial.

At each subsequent weekly visit, the ear was inspected. Each bead was removed after 14 days, or earlier if inflammation was present, and fresh bead(s) placed in the opposite ear, using the same location(s). Beads were worn throughout the period of attendance of groups (i.e. the first 4 weeks of withdrawal) and removed at the last attendance.

### Outcomes

The proposed outcomes to measure the intended effect of acupressure were NRT consumption and nicotine withdrawal symptoms.

We aimed to measure consumption of NRT (patch, gum, microtablets, lozenges or inhalator) in two ways: both from counting used packs at each attendance,[20] and from reports in daily diaries. We assessed withdrawal symptoms using the seven-item Mood and Physical Symptoms Scale (MPSS) [21,22]. The MPSS is sensitive to changes resulting from abstinence [23]: smokers rate the severity of depression, irritability, restlessness, hunger, poor concentration, anxiety and insomnia. We also asked them to rate cravings with the questions 'time spent in urge to smoke' and 'strength of urge to smoke'. We asked one additional question to rate severity of constipation, as discussed by one author [23]. All these measures used five-point scales. Since withdrawal symptoms were an important focus of the study, we asked participants to score the diary daily. Initially, we asked participants to complete the MPSS for just three days in the week before quit date, as recommended. However, when one participant continued to do this for the following week, we altered our procedures to ask participants to complete the diary for seven days for all five weeks of the trial period.

The planned secondary outcomes were: quit rate at four weeks defined as self-reported cessation, validated by carbon monoxide concentration in expired air of ≤ 9 ppm, obtained by clinic staff using the Bedfont Smokerlyser (Bedfont Scientific Ltd, Bedfont House, Holywell Lane, Upchurch, Kent UK) calibrated at the start of trial; and how much discomfort the beads caused (five point scale from none to extreme) recorded in the daily diary. We also asked participants to record how many times they stimulated the beads over 24 hours (in five categories: none, < 5, 5–9, 10–20 and over 20 times), on the premise that a minimum of 5 times would be necessary in the first two weeks unless the bead was already causing discomfort.

At each clinic attendance, participants were interviewed by either AW or RM. They were asked whether they had experienced any adverse events during the previous week that might have been due to NRT or acupressure. This information and CO reading were recorded in a clinic data sheet.

### Analysis

Data were entered into Excel spreadsheets by clinic staff with reference to the usual clinic records where necessary e.g. for missing CO readings.

For the analysis of outcomes, missing data were handled as follows: for NRT use, blank cells indicating possible missing data were completed or not according to the context, which was usually daily application of a nicotine patch. For MPSS, we could not find published guidelines, and none were available from the author (Robert West, personal communication) so we established the following rules: from the seven questions on each daily diary, if three responses or less were missing they were substituted by the mean of the remainder; if more than three responses were missing, that day's diary was discarded. For the remaining outcome measures, no data substitutions were made.

Analysis was descriptive and exploratory, using Excel and SPSS. NRT use was calculated as the weekly mean of the daily total mg of nicotine, using the pack data (e.g. 15 mg for each nicotine 15 mg patch) for all products except for the inhalator for which we used a reasoned estimate of 6 mg, in the absence of published data. MPSS scores were total daily scores averaged for each week. Urge and constipation scores were analysed separately. Analysis was planned per protocol, as we would choose for an efficacy study.

## Results

**Figure 1:**
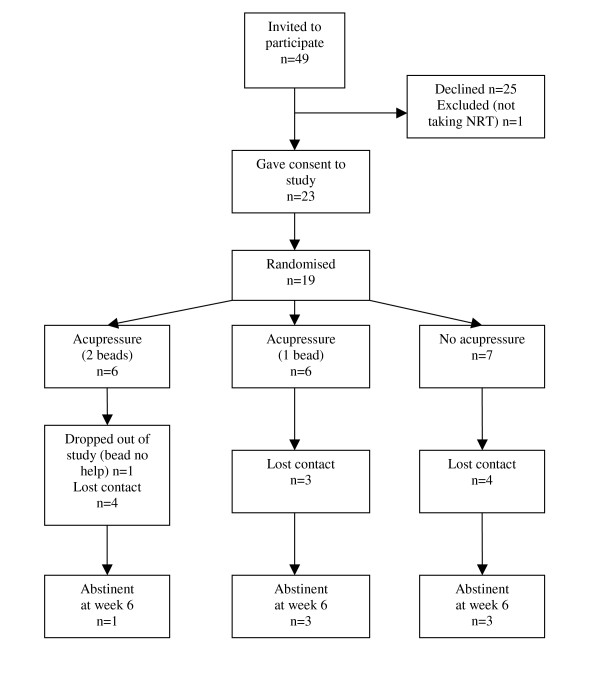
CONSORT diagram of patient flow through the study.

With regard to accrual and dropouts, just under half the smokers who attended four clinics (24 from 49) agreed to participate and were randomised on the quit date. One decided against using NRT so was excluded, and four did not attend the following week so were presumed not to have quit. Thus, 19 (39% of those invited) remained in the study for at least one week after quitting, and 7 (14%) remained throughout the study period (Figure 1). One participant withdrew from the study at week 3 for lack of perceived effect of acupressure, but continued to attend the clinic.

Participants who dropped out of the study were slightly older (47.5, SD 11.5 against 41.7, SD 14.0 years), and reported median 2 previous quit attempts, compared with 6 in those who remained (z = 2.175, p = 0.030, Mann-Whitney U test). Dropouts did not report higher daily consumption of cigarettes, and were similar in nicotine dependence scores, measured expired CO concentration and prescribed form of NRT.

One participant (in group A) did not report pressing the beads more than four times per day from the quit day onwards, but was the only person to rate discomfort as higher than 'slight' at the end of the first week. The other participants reported pressing the bead more than 10 times most days at the beginning of the first week, but only two were continuing at that frequency by the end of the first week. By the end of the second week, only four people reported stimulating it more than five times a day, though one other person reported discomfort. In week quit 3 four participants, and in week quit 4 three participants reported still pressing the bead more than five times a day. Discomfort from the bead was scored as 'none' or 'slight' at the great majority of time points, though two participants, both in group A, reported 'somewhat' on 3 and 4 days, respectively. There were several technical problems with adhesiveness of the dressing: beads frequently either fell off or moved around on the surface of the ear (numbers not recorded). In response to the question on adverse events, two patients reported 'soreness', but no participant reported removing the bead on account of soreness.

Completion rates for diaries varied for different outcomes. Every participant who attended any clinic returned a diary that was at least partially completed. Out of the 95 weeks' diaries of MPSS data, six days (1%) contained too little information to be included, and 15 other items were missing and required substitution (total missing data = 2.0%). Missing data for urge scores were 1%, and for bead pressure and discomfort 3%. Missing data were highest for the number of doses of NRT used per day, at 10.2%. The majority of data missing referred to the use of either a patch, which could be accurately checked by questioning at the subsequent clinic, or use of the second form of NRT which is designed to be used less frequently so missing data might result in only small errors in the analysis. The alternative method for reporting NRT use – counting of empty packets – was generally unsuccessful as very few participants remembered to bring the packets back.

The exploratory analysis is based on 19 smokers who provided outcome data for at least one week after randomisation, week quit 1. Their background characteristics and summary of NRT interventions are given in Table 2. All CO readings were ≤ 9 ppm except in one participant: on week 3 the reading was 10 ppm, but subsequent readings were 1 and 2 ppm. Therefore we considered all who attended to be successful quitters: at the last attendance, seven had quit, one in group A and three each in groups B and C.

**Table 2:** Background characteristics of three groups as analysed (means except where stated, with SDs in parentheses)

	group A	group B	group C
n	6	6	7
males (n)	4	1	0
age in years	51.0 (7.9)	39.8 (18.2)	44.4 (8.4)
initial expired CO	23.7 (12.3)	17.2 (4.5)	19.5 (7.8)
cigs/day	17.5 (6.1)	16.5 (4.4)	17.5 (3.3)
years smoking	34.3 (6.3)	21.0 (11.4)	24.8 (11.2)
quit attempts	4.8 (6.7)	6.8 (6.9)	3.3 (1.9)
quits last 6 mo	0.5	1.0	0.4
live alone (n)	2	1	0
smoker in home (n)	1	1	1
modified Fagerstrom: max score 10	4.7 (1.6)	4.3 (1.5)	6.0 (2.0)
used NRT patch (n)	6	4	7
used 2 forms of NRT (n)	6	2	6

NRT = nicotine replacement therapy

**Figure 2:**
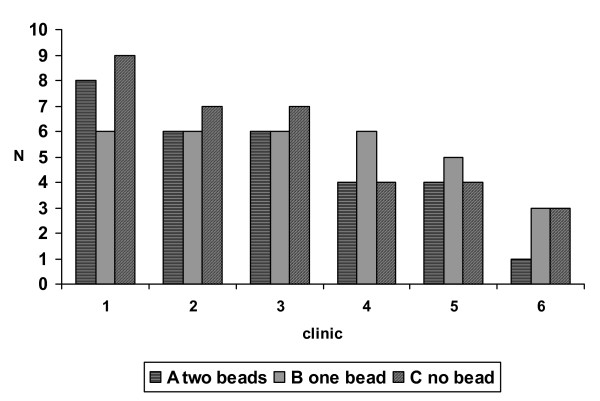
Attendance of 3 groups each week – all attendees were confirmed abstinent on each meeting: all dropouts were assumed to have relapsed.

We limited the comparative analysis to weeks 3 and 5 (see Tables 3 and 4), because of the dropout rate, particularly the small number of participants attending on week 6 as shown in Figure 2. The mean (SD) dose of NRT, based on diary entries, used by participants in the three groups (A, B, C) show no meaningful changes. Mean withdrawal symptom scores (MPSS) are relatively constant throughout the study in all groups (Tables 2 and 3) with no significant difference between group scores on any week (p > 0.5, Kruskal-Wallis for 3 independent groups). Mean MPSS scores for all 19 participants were 11.8 (SD 2.9) and 12.8 (3.0) for the run-in week and quit week respectively. Mean MPSS scores for the first two weeks were not higher in participants who dropped out than those who remained in the study: 11.3 (3.2) and 11.8 (2.8) for the dropouts, and 12.8 (2.2) and 14.5 (2.9) for those who stayed in the study at run-in and quit 1 weeks respectively.

**Table 3:** Mean scores (SD) for main outcomes for participants attending 3^rd ^clinic (first following quit date)

	group A n = 6	group B n = 6	group C n = 7
*Mean NRT consumption*			
Quit week	201.8 (80.4)	126.5 (26.9)	157.9 (32.1)
			
*Mean MPSS scores*			
Run-in week	12.5 (2.9)	11.1 (2.2)	11.9 (3.6)
Quit week	12.2 (3.0)	12.6 (1.8)	13.4 (4.1)

NRT = nicotine replacement therapy

**Table 4:** Mean scores (SD) for main outcomes for participants attending the 5^th ^clinic*

	group A n = 4	group B n = 5	group C n = 4
*Mean NRT consumption*			
quit week	208.8 (56.3)	122.4 (27.9)	179.0 (19.1)
FU1 week	202.5 (36.7)	143.6 (58.7)	162.5 (20.8)
FU2 week	173.3 (27.0)	150.0 (108.2)	152.5 (8.5)
			
*Mean MPSS scores*			
Run-in week	13.1 (1.9)	11.4 (2.4)	12.2 (3.8)
Quit week	11.4 (2.4)	12.9 (1.9)	13.8 (4.0)
FU1 week	12.0 (3.4)	11.8 (3.7)	13.1 (4.7)
FU2 week	12.7 (4.1)	11.2 (3.8)	13.8 (5.1)

*insufficient data to analyse 6^th ^(final) week

NRT = nicotine replacement therapy

FU = follow up

There were no discernible differences between groups for urges or constipation scores (data not presented).

In view of the lack of effects observable in the measured outcomes, the researchers asked some of the participants informally at the end of the study whether they had noticed any discernible effect of the beads. One participant reported that pressing the bead 'definitely helped to relax' her; one reported that 'it was a distraction only' and four reported that the acupressure did not help. Two participants dropped out of the study after reporting a lack of any noticeable effect.

## Discussion

This pilot study found that about half the smokers that were seeking help from a local smoking advice service were willing to try acupressure beads as an adjunct to usual treatment, but the dropout rate during the five week study was 84%. Compliance with instructions to press the bead was good for the first week but then deteriorated. Adverse events were minimal.

Each clinic of approximately 12 smokers generated about five participants in the first week after the quit date, of whom about two remained at the final (sixth) follow up. High dropout rates are not unusual in trials of addiction.

We were surprised how little stimulation the participants reported using, and how little discomfort the beads caused: we designed the study on the premise that acupressure needs to cause a perceptible stimulus for it to be effective. The original observation of an effect of acupuncture in heroin withdrawal involved electrical stimulation of the needles,[6] and other authors have reported using a surgical suture in the ear [24]. Stimulation of indwelling devices is a consistent feature of reports of this method.

Diaries were returned efficiently, but recording of NRT consumption was poor, probably because participants thought we would assume that an NRT patch would be used every day, and because participants found it difficult to recall the use of variable items like gum or inhalator. We noted wide variability in the reported consumption of NRT, particularly in group B. This was due largely to two participants in this group who did not use NRT patches: one used small numbers of microtabs, taking only 16 mg nicotine in the last week: another used large numbers of nicotine gum 4 mg, amounting to 308 mg in the last week. The alternative measure for NRT use, counting used packs, was also unsuccessful. We conclude that further investigation of the best way to measure NRT consumption is needed before this can be considered a suitable outcome for a clinical trial.

In contrast, there were few missing data for MPSS scores. The mean values scored by our participants (ranging between about 11 and 14) are similar to those observed in 83 smokers in a previous study [22]. Daily scores rose dramatically in that study to about 18 among the 43 smokers who voluntarily abstained, without nicotine replacement, for 24 hours. The lack of any such increase in our participants is presumably due to the effects of NRT and the behavioural intervention: either withdrawal symptoms do not occur, or any that do occur are not detected by the MPSS. The lack of any measurable increase in withdrawal symptoms suggests a 'floor effect', i.e. that there is no possibility of measuring an additional effect of acupressure because smoking withdrawal symptoms are already managed effectively within the limits of current measurement tools.

In addition, in interacting with the participants, we detected an initial enthusiasm for trying acupressure at the time of quitting, but a marked lack of enthusiasm for the intervention at the end of the study. Future studies could profitably explore the effectiveness of acupressure in smokers who have chosen acupressure in preference to NRT or bupropion using a patient preference design [25]. High levels of expectation could in themselves increase the effectiveness of acupressure, and may improve compliance with instructions to stimulate the bead. Open studies should be conducted first to determine whether an effect exists, then by blinded studies using a placebo intervention, to test the causal relationship. We have not been able to design a convincing placebo for acupressure beads. Placebo-controlled studies of acupressure in smoking cessation require careful consideration of the ethical aspects: in view of the proven benefits of certain interventions, and the life-threatening consequences of failure, smokers should not be offered only a control intervention which the researcher expects to be ineffective. This is why we initially sought a dose-related effect in our design, and why we offered acupressure as an adjunct to known effective treatment.

## Conclusion

This pilot study suggests that any effects of acupressure on NRT consumption or nicotine withdrawal symptoms in smoking cessation, as an adjunct to the use of NRT and behavioural intervention, are unlikely to be detectable by the methods used in this study, and further preliminary studies are required before the hypothesis can be tested.

## Competing interests

AW receives an income from the British Medical Acupuncture Society as editor in chief of their journal, *Acupuncture in Medicine*. AW also runs a private acupuncture clinic, but does not offer acupuncture for smoking cessation. RM and JC declare that they have no competing interests.

## Authors' contributions

All authors were involved in the design of the study, and writing and editing the report: AW and RM collected the data; AW performed the analysis. All authors read and approved the final manuscript.

## Pre-publication history

The pre-publication history for this paper can be accessed here:


